# Supplementary material for: Multistability and dynamic transitions of intracellular Min protein patterns
Source: Mol Syst Biol. 2016 Jun 8;12(6):873. doi: 10.15252/msb.20156724 (PMC4923923; doi:10.15252/msb.20156724)
Supplement: Supplementary file 1 — Appendix [file MSB-12-873-s001.pdf]

1 Numerical stability analysis of Min oscillation patterns

This section describes how the stability of Min oscillation patterns in rectangular-shaped 3D cell geometries is determined numerically.
We analyse the stability with respect to spatial variations in the MinD attachment rate. For the sake of simplicity such variations will be restricted to linear profiles of the attachment rate k_D,

$$k_D(x, y, z) = \bar{k}_D \left(1 + 2s \frac{x \cos a + y \sin a}{|l_x \cos a + l_y \sin a|}\right). \quad (1)$$

Here l_x and l_y denote cell length and width ($|x| < l_x/2$, $|y| < l_y/2$), $\bar{k}_D$ the mean attachment rate, s characterizes the slope of the profile ($0 \leq s \leq 1$) and a denotes the direction of rate variation with respect to the x-axis ($0 \leq a \leq \pi/2$).

In order to investigate how the stability of oscillation patterns depends on cell size, geometry and the MinD recruitment rate k_{dD}, we define 18 different rectangular-shaped cell bodies with varying length and width, but identical height $2r$. The cell lengths were increased from $4\mu m$ to $10\mu m$, the cell width from $3\mu m$ to $5\mu m$, both in steps of $1\mu m$. For each of these geometries, we perform the following procedure independently for all MinD recruitment rates $k_{dD} \in \{0.03, 0.035, \ldots 0.1\mu m^3/s\}$:

1. The system is prepared in a transversal oscillation pattern in y-direction by solving the nonlinear equations (c.f. Materials and Methods) numerically for 200s with an attachment rate variation in y-direction using parameters $s = 1$, $a = \pi/2$ in Equ. (1).

2. Subsequently the spatial variation of k_D is removed by setting $s = 0$ and the pattern is observed for a simulation time of 2000s with spatially homogeneous attachment rate.

   We then determine if the pattern remains stable during this period by comparing the evolution of the concentration u_{DD} at two points $\mathbf{p_1}$ and $\mathbf{p_2}$ in the cell interior which are located axisymmetrically with respect to the y-axis. We here choose $\mathbf{p_1} = [0.95(-lx/2 + r), 0, r/2]$ and $\mathbf{p_2} = [0.95(lx/2 - r), 0, r/2]$.
   If the pattern is stable, $u_{DD}(\mathbf{p_1}, t)$ and $u_{DD}(\mathbf{p_2}, t)$ will, apart from small numerical errors, not deviate from each other. However, if the pattern exists only transiently and switches into a longitudinal oscillation, the concentration in $\mathbf{p_1}$ and $\mathbf{p_2}$ will loose its symmetry and the

signals will deviate.
We therefore apply the following criterion for stability:

$$\max_{t\in\{0,100s,\dots,2000s\},i\in\{1,2\}} \left\{ \frac{|u_{DD}(\mathbf{p_1},t) - u_{DD}(\mathbf{p_2},t)|}{u_{DD}(\mathbf{p_i},t)} \right\} \begin{cases} \geq \alpha \Rightarrow & \text{pattern not stable} \\ < \alpha \Rightarrow & \text{pattern stable} \end{cases} \tag{2}$$

Eq. 2 states that a pattern is only considered stable if the maximum relative deviation of the concentration u_{DD} between $\mathbf{p_1}$ and $\mathbf{p_2}$ over the solution interval does not exceed a threshold α. By inspection we find that $\alpha = 0.05$ is a reasonable choice.
An example for the application of Eq. (2) is shown in Fig. 1.

3. In the case that a stable pattern is found in the previous step, we use its final system state as initial configuration for independent simulations in which the stability is tested against perturbations given by spatial attachment rate variations with different slopes s and directions a (cf. Eq. (1)).

   Specifically, for each combination

   $$(s,a) \in \{0.2, 0.4, \dots 1.0\} \times \{0, pi/18, \dots, pi/2 - pi/18\}$$

   we compute the solution for an interval of 2000s and determine the type of the final pattern. We here distinguish between three different pattern types: transversal pole-to-pole oscillation, longitudinal pole-to-pole oscillation and longitudinal stripe-shaped oscillations. The following recipe describes how this evaluation is automated:

   - The three pattern types are distinguishable by their different spatial symmetries. We therefore measure and compare the values of all concentrations $u \in \{u_{DD}, u_{DT}, u_E\}$ on 6 different straight lines located in the cell interior (cf. Fig. 2) :

$$\begin{aligned} \gamma_1 &= \{(x,y,r/2) : x = -0.95(l_x/2 - r) &\wedge |y| \leq 0.95(l_y/2 - r)\}, \\ \gamma_2 &= \{(x,y,r/2) : x = 0 &\wedge |y| \leq 0.95(l_y/2 - r)\}, \\ \gamma_3 &= \{(x,y,r/2) : x = 0.95(l_x/2 - r) &\wedge |y| \leq 0.95(l_y/2 - r)\}, \\ \eta_1 &= \{(x,y,r/2) : |x| \leq 0.95(l_x/2 - r) &\wedge y = -0.95(l_y/2 - r)\}, \\ \eta_2 &= \{(x,y,r/2) : |x| \leq 0.95(l_x/2 - r) &\wedge y = 0.95(l_y/2 - r)\}. \end{aligned} \tag{3}$$

- Using the notation $\langle u\rangle_\gamma \equiv |\gamma|^{-1}\int_\gamma u\mathrm{d}s$, we define the following three measures for the symmetry of the pattern, again for all $u \in \{u_{DD}, u_{DT}, u_E\}$:

$$\begin{aligned} p_L(u) &= \frac{|\langle u\rangle_{\gamma_1} - \langle u\rangle_{\gamma_3}|}{\langle u\rangle_{\gamma_1+\gamma_3}}, \\ p_T(u) &= \frac{|\langle u\rangle_{\eta_1} - \langle u\rangle_{\eta_2}|}{\langle u\rangle_{\eta_1+\eta_2}}, \\ p_S(u) &= \frac{|\langle u\rangle_{\gamma_1+\gamma_3} - \langle u\rangle_{\gamma_2}|}{\langle u\rangle_{\gamma_1+\gamma_2+\gamma_3}}. \end{aligned} \tag{4}$$

  The line integrals in $\langle u\rangle_\gamma$ are here approximated by the value of u at three points, the two endpoints and the midpoint of the line γ. The concentrations are evaluated at the last time step, $t = 2000s$. In case of a longitudinal pole-to-pole oscillation, the signals at γ_1 and γ_3 oscillate in opposition, resulting in a large p_L. Similarly, transversal pole-to-pole oscillations yield large values of p_T. Finally, in case of a longitudinal stripe pattern (with two nodes of oscillation) the concentrations at γ_1 and γ_3 are in phase with each other and in opposition with the signal at γ_2. Therefore large values of p_S characterize longitudinal stripe oscillations.

- Taken these considerations together, we conclude that the type of the oscillation pattern can be determined reliably by the following rule:

$$\text{if} \quad p(u) = \max\{p_L(u), p_T(u), p_S(u)\} = \begin{cases} p_L(u) & \Rightarrow \quad \text{long. pole-to-pole} \\ p_T(u) & \Rightarrow \quad \text{trans. pole-to-pole} \\ p_S(u) & \Rightarrow \quad \text{long. stripe.} \end{cases} \tag{5}$$

  To obtain a single, final result for the pattern type we take the vote over the independent evaluations for $u \in \{u_{DD}, u_{DT}, u_E\}$. The unspecified case that the three concentrations yield all three different pattern types did not occur.

For fixed cell geometry, this procedure firstly provides us with a threshold for the MinD recruitment rate k^*_{dD} above which the initiated transversal oscillation persists in step 2. Secondly, for each $k_{dD} > k^*_{dD}$ one obtains thresholds in direction and slope of the perturbation above which the pattern switches.

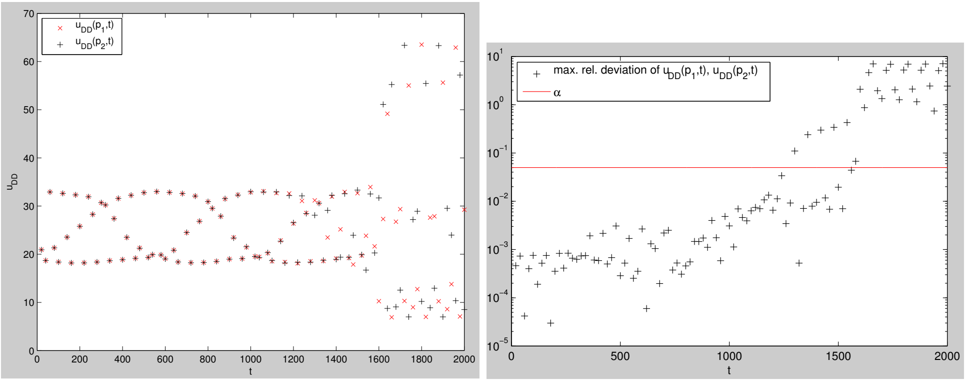

(a) (b)

Figure 1: Example for a transversal oscillation pattern that is found unstable using Eq. (2). The concentrations $u_{DD}(\mathbf{p_1}, t)$ and $u_{DD}(\mathbf{p_2}, t)$ deviate considerably at $t \approx 1600s$, since the pattern changes into a longitudinal oscillation (**a**). The relative deviation therefore exceeds the threshold α and the pattern is found unstable (**b**). Parameters: $l_x = 8, l_y = 3, k_{dD} = 0.07\mu m^3/s$

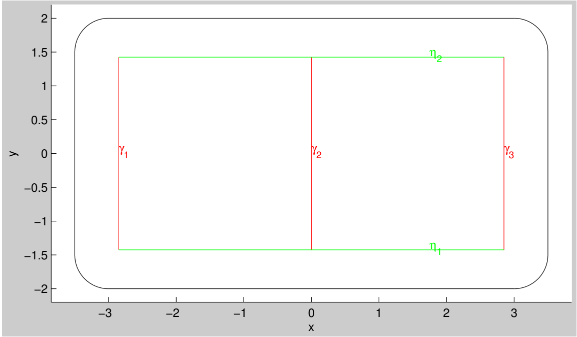

Figure 2: Illustration of the straight lines along which u_{DD} is evaluated in order to determine the type of the final oscillation pattern. The contour indicated in black is the shape of a cell with parameters $l_x = 7$ and $l_y = 4$ in topview. The straight lines γ_i and η_i for pattern determination are indicated in red and green, respectively. The position of the lines in z-direction is $z = r/2$.

Interchanging the role of cell length and cell width enables us analyse the stability of longitudinal oscillations analogously to steps 1 to 3. Since all non-perturbed longitudinal oscillations are found stable in step 2 for the

considered range of k_dD, no critical recruitments rates are determined in this case.

The numerical solution of the model equations (c.f. Materials and Methods) was computed using *Comsol Multiphysics 4.4*. To reduce the number of necessary simulations for the parametric sweep in step 3, we utilised the observation that the stability of a pattern decreases with the slope of the perturbation and (partly) skipped parameter sets where the stability or destability could be inferred from previous runs.

2 Characterization of Min oscillation patterns

The goal of this study is to investigate how the occurrence of specific types of oscillation patterns depends on cell geometry and MinD recruitment rate k_{dD}. As opposed to the previous section, the Min system is not prepared in a specific solution. Instead, the initial condition is a spatially homogeneous configuration.

We again impose a linear variation of the attachment rate k_D with different slopes and directions according to Eq. (1). The solution is calculated for $0 \leq t \leq 4000s$. We subsequently determine the final pattern that the Min system settles into with the procedure explained in step 3 of section 1, in which we evaluate the concentrations at the last time step $t = 4000s$.

This analysis is performed independently for the cell geometries and MinD recruitment rates reporded in Fig. 4 of the main text, and for parameters $a \in \{0, \pi/10, \ldots, \pi/2\}$ and $s = 0.2$ in the attachment rate profile.

With width specified:

Parameter	Value	Description
D_D	$16\mu m^2 s^{-1}$	Cytosolic Diffusion coefficient MinD
D_E	$10\mu m^2 s^{-1}$	Cytosolic Diffusion coefficient MinE
D_m	$0.013\mu m^2 s^{-1}$	Membrane Diffusion coefficient MinDE
λ	$6s^{-1}$	Cytosolic nucleotide exchange rate
k_{de}	$0.5s^{-1}$	MinDE Detachment rate
k_D	$0.1\mu m s^{-1}$	MinD attachment rate constant
k_{dD}	$0.1\mu m^3 s^{-1}$	MinD recruitment rate constant
k_{dE}	$0.435\mu m^3 s^{-1}$	MinE recruitment rate constant
C_D	$602/\mu m^3$	total MinD density
C_E	$301/\mu m^3$	total MinE density

Table 1: Parameter values used in numerical simulations
