# Supplementary material for: Multistability and dynamic transitions of intracellular Min protein patterns
Source: Mol Syst Biol. 2016 Jun 8;12(6):873. doi: 10.15252/msb.20156724 (PMC4923923; doi:10.15252/msb.20156724)
Supplement: Supplementary file 2 — Expanded View Figures PDF [file MSB-12-873-s002.pdf]

Expanded View Figures

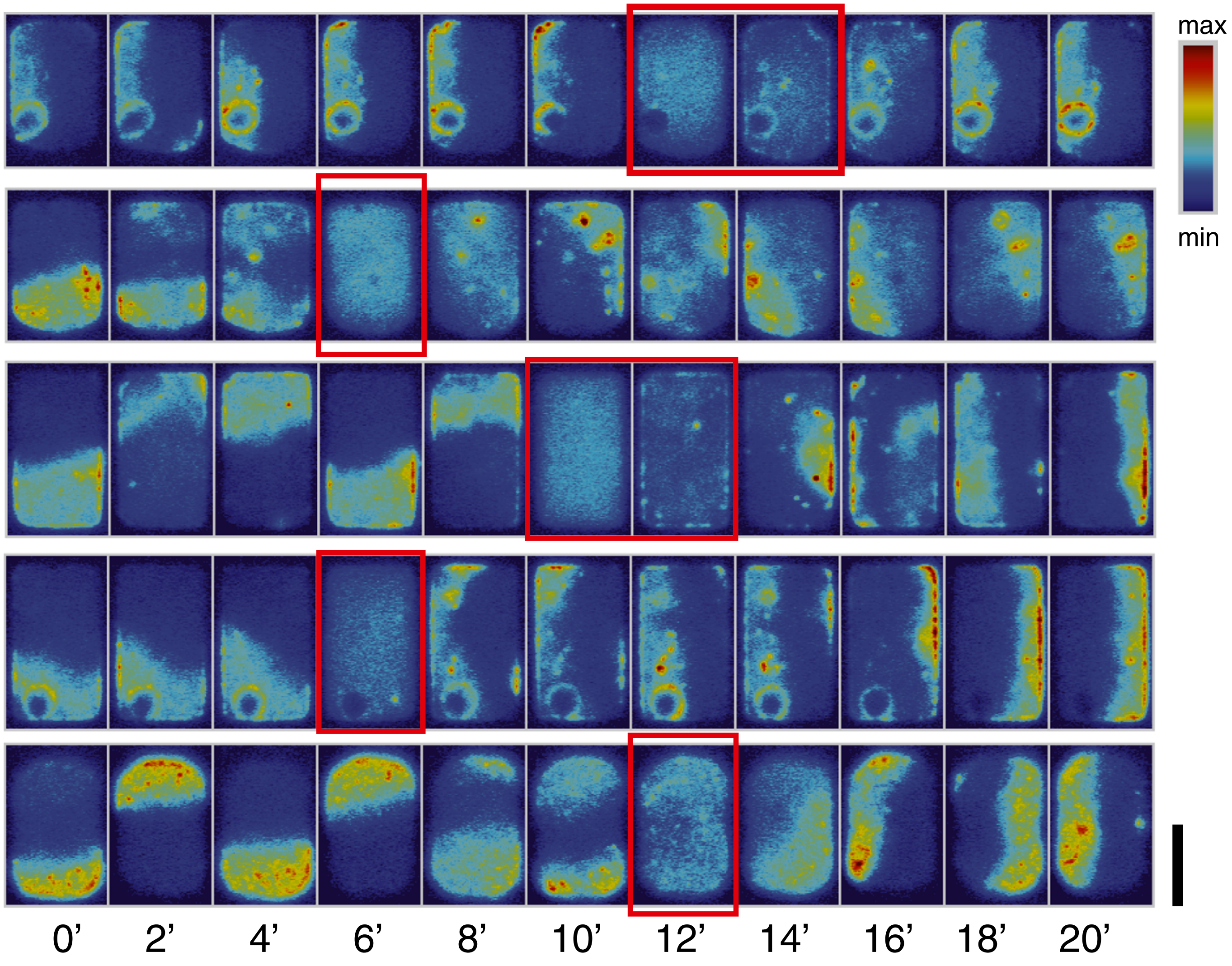

Figure EV1. Disruption and re-emergence of Min patterns in cells of 5 µm in width.
The red boxes show the near-homogeneous state. The color scale indicates MinD concentration. Scale bar = 5 µm.

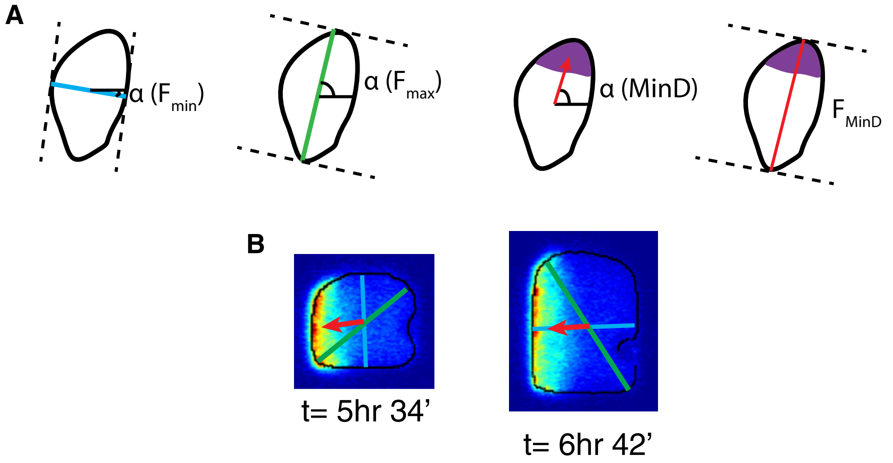

Figure EV2. Illustrations of maximum/minimum Feret diameters.

A From left to right showing the minimum Feret diameter and its angle, the maximum Feret diameter and its angle, the angle of the MinD polar zone, and the Feret diameter corresponding to this angle.

B Two examples of Feret diameters and angles in the cell shown in Fig 5D–F.

 © 2016 The Authors
