# Supplementary material for: Multistability and dynamic transitions of intracellular Min protein patterns
Source: Mol Syst Biol. 2016 Jun 8;12(6):873. doi: 10.15252/msb.20156724 (PMC4923923; doi:10.15252/msb.20156724)
Supplement: Supplementary file 3 — Video EV1 [file MSB-12-873-s003.zip › 6724_VideoEV1/Video_EV1_legend.docx]

**Video EV1. Disruption and re-emergence of Min patterns in cells of 5 μm in width imaged at 2-min intervals.**
