# Supplementary material for: Multistability and dynamic transitions of intracellular Min protein patterns
Source: Mol Syst Biol. 2016 Jun 8;12(6):873. doi: 10.15252/msb.20156724 (PMC4923923; doi:10.15252/msb.20156724)
Supplement: Supplementary file 4 — Video EV2 [file MSB-12-873-s004.zip › MSB_6724_VideoEV2/Video_EV2_legend.docx]

**Video EV2. Robust transversal oscillations imaged at 20-sec intervals.**
