# Supplementary material for: Multistability and dynamic transitions of intracellular Min protein patterns
Source: Mol Syst Biol. 2016 Jun 8;12(6):873. doi: 10.15252/msb.20156724 (PMC4923923; doi:10.15252/msb.20156724)
Supplement: Supplementary file 5 — Video EV3 [file MSB-12-873-s005.zip › MSB_6724_VideoEV3/Video_EV3_legend.docx]

**Video EV3. Co-imaging of sfGFP-MinD and MinE-mKate2 during a symmetry-breaking process.**
