# Supplementary material for: Multistability and dynamic transitions of intracellular Min protein patterns
Source: Mol Syst Biol. 2016 Jun 8;12(6):873. doi: 10.15252/msb.20156724 (PMC4923923; doi:10.15252/msb.20156724)
Supplement: Supplementary file 6 — Video EV4 [file MSB-12-873-s006.zip › MSB_6724_VideoEV4/Video_EV4_legend.docx]

**Video EV4. Time evolution of patterns in cells that adopt different pattern modes due to different constraints on their growth, imaged at 2-min intervals.**
