# Supplementary material for: Multistability and dynamic transitions of intracellular Min protein patterns
Source: Mol Syst Biol. 2016 Jun 8;12(6):873. doi: 10.15252/msb.20156724 (PMC4923923; doi:10.15252/msb.20156724)
Supplement: Supplementary file 7 — Video EV5 [file MSB-12-873-s007.zip › MSB_6724_VideoEV5/Video_EV5_legend.docx]

**Video EV5. An example of stochastically switching Min patterns in cells with low aspect ratios.**
