# Supplementary material for: Multistability and dynamic transitions of intracellular Min protein patterns
Source: Mol Syst Biol. 2016 Jun 8;12(6):873. doi: 10.15252/msb.20156724 (PMC4923923; doi:10.15252/msb.20156724)
Supplement: Supplementary file 8 — Video EV6 [file MSB-12-873-s008.zip › MSB_6724_VideoEV6/Video_EV6_legend.docx]

**Video EV6. Various examples of pattern transitions in cells with different dimensions.**
